# Variation of Maximum Tree Height and Annual Shoot Growth of Smith Fir at Various Elevations in the Sygera Mountains, Southeastern Tibetan Plateau

**DOI:** 10.1371/journal.pone.0031725

**Published:** 2012-03-01

**Authors:** Yafeng Wang, Katarina Čufar, Dieter Eckstein, Eryuan Liang

**Affiliations:** 1 Key Laboratory of Tibetan Environment Changes and Land Surface Processes, Institute of Tibetan Plateau Research, Chinese Academy of Sciences, Beijing, China; 2 Department of Wood Science and Technology, Biotechnical Faculty, University of Ljubljana, Ljubljana, Slovenia; 3 Division Wood Biology, Department of Wood Science, University of Hamburg, Hamburg, Germany; 4 Graduate University of Chinese Academy of Sciences, Beijing, China; University College London, United Kingdom

## Abstract

Little is known about tree height and height growth (as annual shoot elongation of the apical part of vertical stems) of coniferous trees growing at various altitudes on the Tibetan Plateau, which provides a high-elevation natural platform for assessing tree growth performance in relation to future climate change. We here investigated the variation of maximum tree height and annual height increment of Smith fir (*Abies georgei* var. *smithii*) in seven forest plots (30 m×40 m) along two altitudinal transects between 3,800 m and 4,200/4,390 m above sea level (a.s.l.) in the Sygera Mountains, southeastern Tibetan Plateau. Four plots were located on north-facing slopes and three plots on southeast-facing slopes. At each site, annual shoot growth was obtained by measuring the distance between successive terminal bud scars along the main stem of 25 trees that were between 2 and 4 m high. Maximum/mean tree height and mean annual height increment of Smith fir decreased with increasing altitude up to the tree line, indicative of a stress gradient (the dominant temperature gradient) along the altitudinal transect. Above-average mean minimum summer (particularly July) temperatures affected height increment positively, whereas precipitation had no significant effect on shoot growth. The time series of annual height increments of Smith fir can be used for the reconstruction of past climate on the southeastern Tibetan Plateau. In addition, it can be expected that the rising summer temperatures observed in the recent past and anticipated for the future will enhance Smith fir's growth throughout its altitudinal distribution range.

## Introduction

Environmental factors determine the structure, dynamics, and species composition of forest communities [1]–[4]. Tree growth may be subjected to environmental gradients (in particular temperature gradients) associated with elevation, providing convenient scenarios to investigate the potential responses of forest growth in natural environments to future climate change, via a space-for-time substitution [5], [6]. There are two main useful indicators for examining the responses of tree growth to climate variability: ring width and the annual height increment of trees. Due to the easier access of tree-ring width data in comparison to height increment time series, the radial growth of trees along altitudinal gradients has been the topic of many dendroecological studies (see a brief summary by Liang et al., 2010) [7]. However, shoot growth and its response to climate along elevational gradients have received less attention [8].

Shoot extension of most coniferous species is a two-year process involving the formation of terminal buds during the previous year and shoot elongation in the current year [9]–[11]. Several studies have shown that at high latitudes or altitudes, shoot elongation of the current year is mainly controlled by the mean July temperature of the previous year [12]–[14], but there have also been reports of significant positive effects of current June or July temperatures on shoot elongation [8], [14]. Shoot growth of trees is regarded as a result of numerous inter-correlated ecological factors (e.g., nutrient availability, precipitation, sunshine duration, wind intensity) but the sensitivity of shoot extension to temperature is generally considered to be most important for trees at high altitudes or latitudes [15], [16]. To date, the variation in tree height and height growth along temperature gradients has in our opinion received less attention than it deserves.

Maximum tree height is an important indicator for understanding several properties of plant communities, including total standing biomass and resource use [4], [17]. Since the upper tree line is identified by tree height [18], the variation in maximum tree height along a species' altitudinal distribution provides a quantitative baseline for understanding patterns of resource use, spatial structure and demography and, hence, the physiological mechanism of alpine tree-line formation, in particular on the southeastern Tibetan Plateau, where the natural tree line reaches one of the highest altitudes in the world.

Smith fir (*Abies georgei* var. *smithii*) forests grow at elevations from 3,550–4,400 m in the Sygera Mountains on the southeastern Tibetan Plateau. It can therefore be taken as a model tree species for investigating variation in maximum tree height and tree growth along altitudinal gradients. It has been shown that the radial growth of Smith fir at different elevations is mainly controlled by mean summer (particularly July) minimum temperature [7]. However, little is known about the height increment of this species and its response to climate along its elevational distribution range. In addition, recent studies have revealed that climate warming has been causing rapid glacier retreat, increasing recruitment and radial growth of trees at tree lines and timberlines on the Southeast Tibetan Plateau [19]–[22]. It remains unknown how the height growth of trees in different altitudinal belts of the Tibetan Plateau is responding to recent abnormal warming.

The objectives of this study on Smith fir from various elevations and exposures in the Sygera Mountains were to (1) detect the site-dependent variability of maximum/mean tree height and of annual height increments, (2) explore the dominant environmental factors limiting height growth, and (3) reveal whether climate warming enhances height growth of Smith fir in a specific altitudinal belt. We hypothesized that the limiting effect of temperature on height growth would increase with increasing elevation. Based on the results from the radial growth of Smith fir at various elevations, height growth could be more sensitive to climate of the current year in a comparison with the previous years.

## Materials and Methods

### Ethics Statement

We do not require an ethics statement because the research topic focuses on tree growth in natural public forests.

### Study area and climate

**Figure 1:**
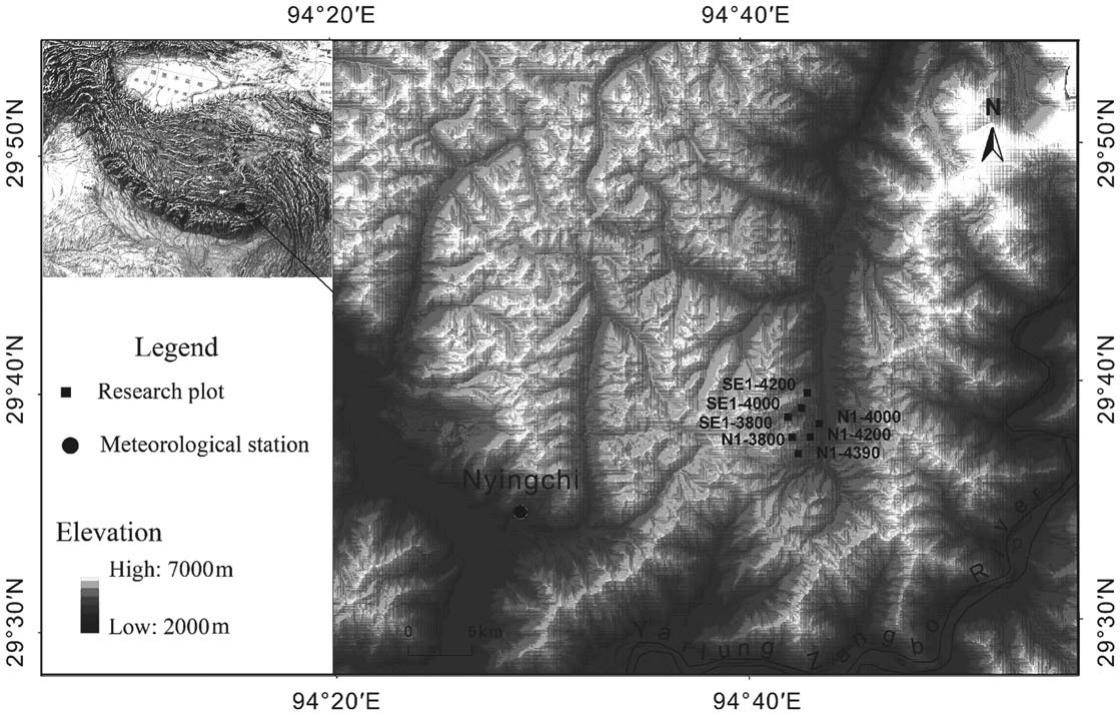
The location of the Sygera Mountains on the Tibetan Plateau, and topographic map showing the location of the seven research plots and the meteorological station at Nyingchi, southeastern Tibet, China. SE1 and N1 represent southeast- and north-facing slopes, respectively; numbers (3800, 4000, 4200, 4390) denote the elevation in m a.s.l.

The study area, located in the eastern Sygera Mountains (29°10′–30°15′N, 93°12′–95°35′E) (Fig. 1), is influenced by the south Asian monsoon along the Yarlung Zangbo River Valley, resulting in abundant summer rainfall. At the meteorological station in Nyingchi (29°34′N, 94°28′E, 3,000 m a.s.l), a mean annual precipitation from 1960 to 2009 of 672.6 mm was recorded, 71.8% of which is received from June till September. The warmest and the coldest months are July (mean temperature of 15.9°C) and January (0.6°C), respectively [7]. The records show significantly increasing trends in mean and minimum temperatures for all seasons since the 1960 s [19]. In contrast, increasing trends in maximum temperatures have been restricted to the spring (March-May) and winter (December-February) seasons. Additionally, an increasing trend in annual and summer precipitation is evident.

### Study sites

Along the two studied altitudinal transects, located on the eastern side of the Sygera Mountains, the natural vegetation composition shows clear changes with increasing elevation. Depending on the local topography and slope aspect, the tree line of Smith fir varies from 4,250 to 4,400 m a.s.l., where the annual precipitation ranges from 750 to 960 mm as observed from 2007 to 2009 [23], [24]. In general, it is pure Smith fir forest with various species in the understory; mainly Smith fir and *Lonicera* spp. from 3,700–4,000 m a.s.l, Smith fir and lichen (*Actinothuidium* spp.) communities from 4,000–4,320 m a.s.l., while Smith fir and *Rhododendron* spp. communities are dominant from 4,320–4,400 m a.s.l. and alpine shrubs and meadows are widely distributed above 4,400 m [25].

We selected seven forest plots, four with a northern and three with a southeastern exposure (Fig. 1). Each plot was 30 m×40 m in size and situated in a topographically uniform area, with the longer side of the plot parallel to the altitudinal gradient. The plots were given the codes N1-3800, N1-4000, N1-4200, N1-4390 and SE1-3800, SE1-4000, SE1-4200, where N1 and SE1 represent north- and southeast-facing slopes and the numbers indicate the elevation. Little evidence of human disturbance was found in any of the sampling stands.

Smith fir also grows at lower altitudes but the distribution ranging from 3,550–3,800 m a.s.l. was not included in our study because it has been disturbed by anthropogenic interference.

### Tree selection and measurements

We counted the total number of trees on each of the seven plots. Their height was only measured if they were more than 3 years old, which was determined by the number of branch whorls. Tree height was obtained using a measuring stick if the tree was shorter than 2 m or with a clinometer if the tree was taller than 2 m.

We selected 25 trees per plot (altogether 175 trees) to measure the annual height increment along the main stems based on bud scars (internodes or branch whorls) [8], [26]–[28], with an accuracy of ±0.1 cm. Trees with a height between 2 and 4 m were targeted for measurement, since it was considered that trees less than 2 m in height would be too young for our research topic and it would be difficult to measure trees higher than 4 m. Similar to other studies [8], [26], [27], the increment was measured from the uppermost height increment, i.e., from the sampling year (2009) downwards, until intense bark formation hampered the observation of bud scars.

The diameter at breast height (DBH) was measured in all trees higher than 1.3 m. Two increment cores, one at breast height and one at the stem base, were taken from trees whose DBH exceeded 5 cm. The cores from the base were processed following standard dendrochronological practice in order to obtain the tree age [29]. The age of juvenile trees was estimated non-destructively in the field by counting the terminal bud scars (internodes or branch whorls) along the main stem.

### Development of height increment chronologies

Height increment series with a similar variation through time were cross-dated similar to tree-ring width data and checked using the COFFCHA program [30]. Deviating height increment series were excluded because they were probably affected by non-climatic factors, such as between-tree competition. Because of the shortness of the individual height-increment series, they were not standardized before averaging them to site-specific height-increment chronologies.

### Data analysis

On each plot, the maximum tree height was used to identify the height variation along the two altitudinal gradients. Furthermore, the mean tree height was calculated from the 20 highest trees on each plot. We thus excluded the effect of seedlings and juveniles, which predominated on all plots.

Height increment data (of 25 trees per plot) were used to calculate the mean annual height growth rate over a common period (1991–2009). On each plot, the relationships among height, age and DBH were also investigated. One-way ANOVA was employed to test the differences in annual height growth rate of Smith fir along the two altitudinal transects.

In order to determine the climatic driving factors for tree height growth, correlations were computed for the period 1961–2009 between each height increment chronology and monthly climate variables from the previous September to the current July. Monthly sums of precipitation, monthly mean, maximum and minimum temperatures were from the meteorological station in Nyingchi, where the variations of climatic records are confident indicators of the conditions at higher elevations [31].

## Results

### Height growth vs. altitude

**Figure 2:**
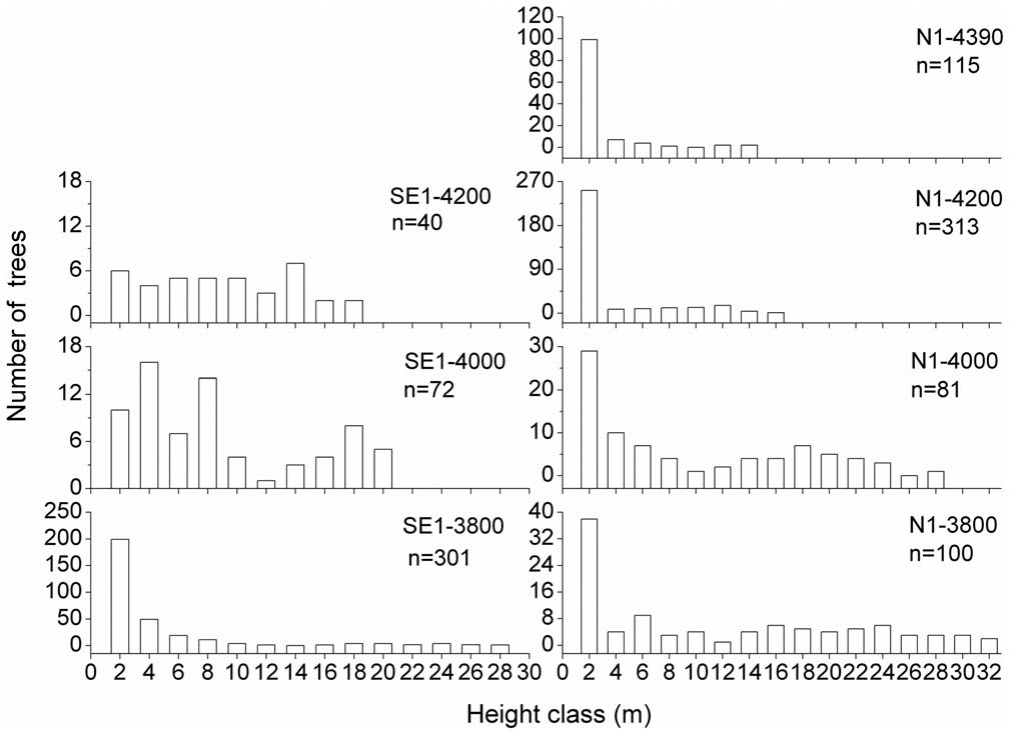
Number of trees per height class along the two altitudinal gradients; n, number of trees per plot.

**Figure 3:**
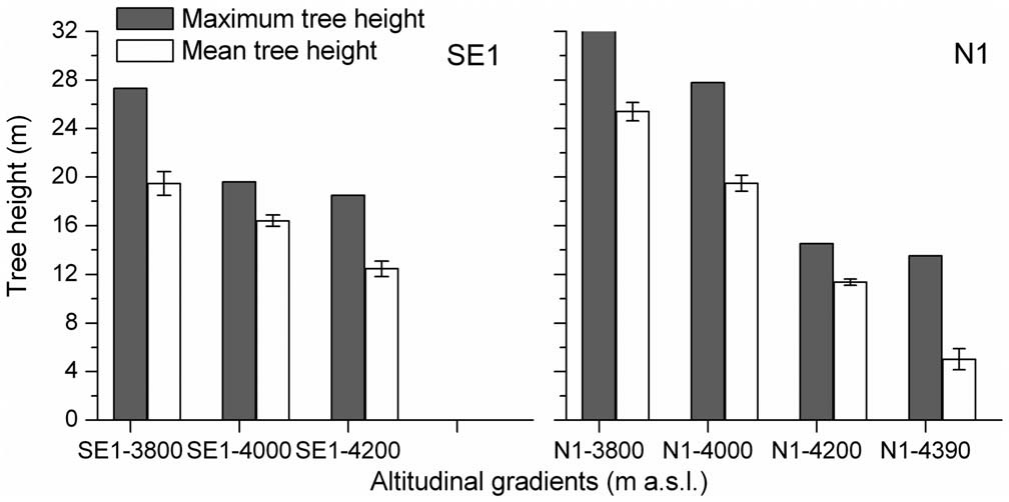
Maximum tree height and mean height (with standard deviation) of the 20 highest trees per plot along the two altitudinal gradients.

On 5 out of 7 plots, the largest number of trees was in the lowest height classes; on the south-facing slope, this applied only for the lowest plot (Fig. 2). In any case, maximum tree height decreased with increasing altitude; the mean height of the highest 20 trees also followed this trend along both transects (Fig. 3). Due to a limit of our plot size and sparse tree density at higher elevations, it is likely that maximum tree height was not exactly captured.

**Figure 4:**
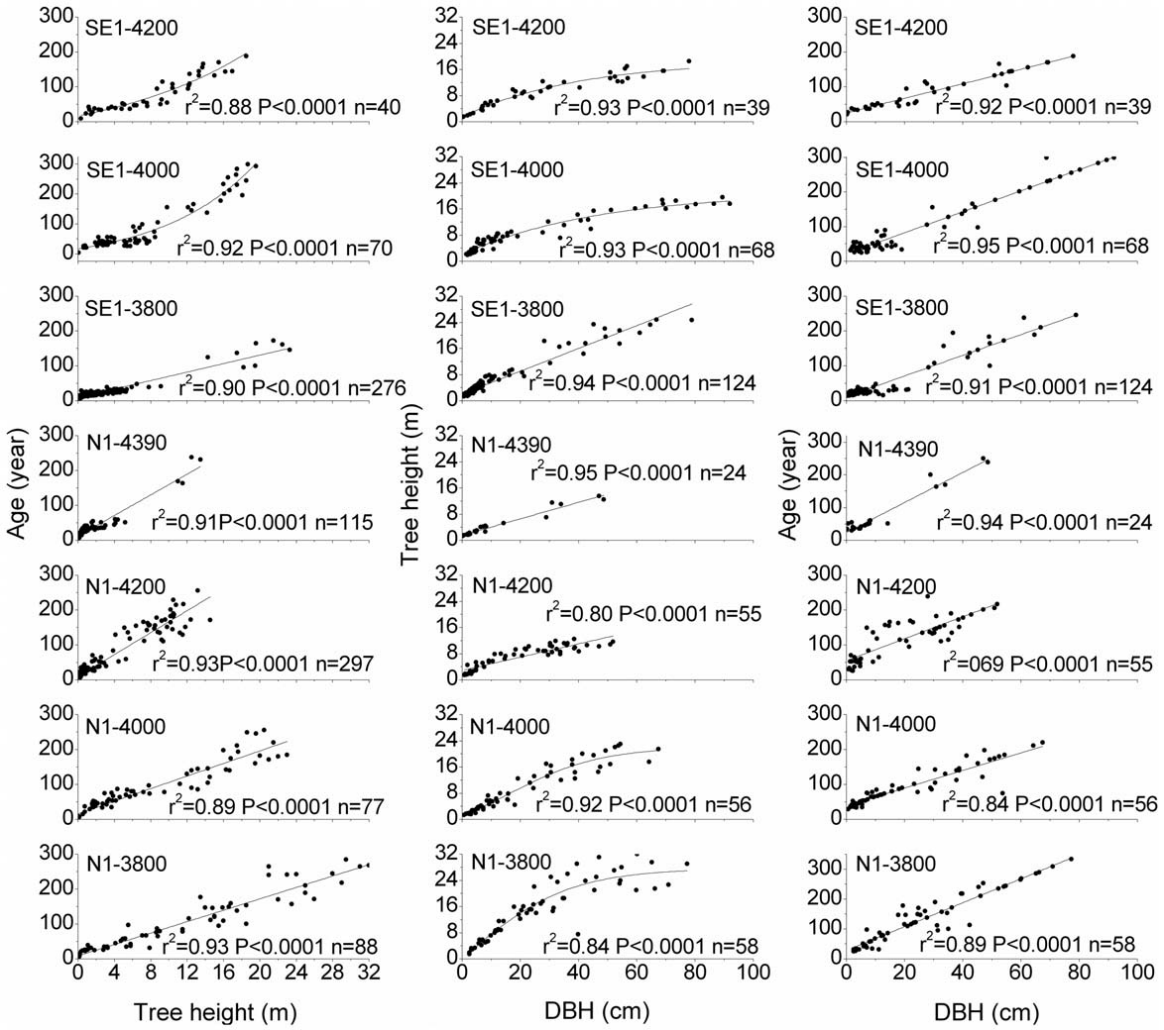
Linear or nonlinear relationships among height, DBH and age of the trees along the two altitudinal gradients.

Highly significant correlations existed among height, age and DBH of the trees along both altitudinal gradients (Fig. 4), with coefficients from 0.8–0.95 (p<0.01). Briefly, this means that the older the trees, the thicker and higher they are. It should be mentioned, however, that the tree height/DBH relationship may follow a non-linear saturation function (as shown in some cases), particularly when the age range of the trees included is large enough.

**Figure 5:**
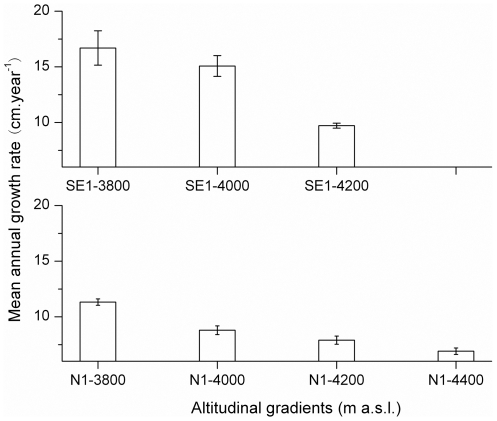
Mean annual height-growth rate of the trees from 1991–2009 along the two altitudinal gradients.

The mean annual height growth rates (cm/year) from 1991–2009, which is the common period for all stands, were higher on the southeast-facing than on the north-facing slope, as well as higher at low elevations (16.7 and 11.32 cm/year for SE1-3800 and N1-3800, respectively) and lower at high elevations (9.7 and 6.9 cm/year for SE1-4200 and N1-4390, respectively) (Fig. 5). According to one-way ANOVA analysis, the annual height-growth rate along the two altitudinal transects is significantly different between the southeastern/northern study plots (SE1: F = 8.02>F0.05 = F_crit_ = 3.17; N1: F = 17.53>F0.05 = F_crit_ = 2.73).

### Height increment series along the altitudinal gradients

Height increment series of different lengths were constructed from 1950–2009; the common period was from 1991–2009. Series within the study plots could be well cross-dated, with mean correlations ranging from 0.45–0.92 (p<0.01). The chronologies were longest at higher elevations, i.e., 4,200 m a.s.l. or higher, and shortest at lower elevations, particularly at 3,800 and 4,000 m a.s.l. on the SE1 slope. Close to the upper timberline, the annual height increments were shorter than at lower elevations.

**Figure 6:**
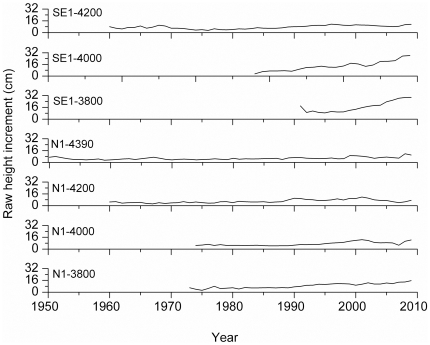
Raw height-increment series along the two altitudinal gradients.

It should be mentioned that the inter-annual fluctuation of height increment was highly consistent among most sites (correlation coefficients from 0.57–0.84, p<0.01; except for a weak correlation between SE1-3800 and SE1-4200). Moreover, a slightly increasing trend in height increment (raw shoot-increment series) was detected on all plots (Fig. 6).

### Correlation between height increment and climatic variables

**Figure 7:**
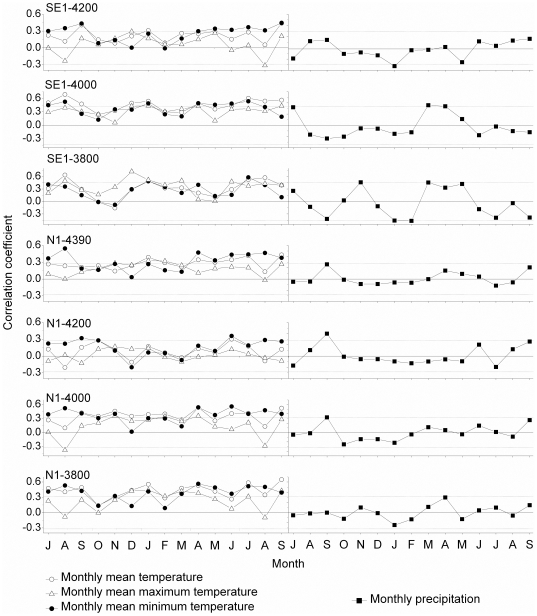
Correlation between the seven height-increment chronologies of Smith fir with climatic variables from July of the previous year to September of the current year, along the two altitudinal gradients; horizontal dotted lines denote 95% confidence limits.

The annual height growth correlated positively with temperature (mainly monthly minimum and mean temperatures) from July-September of the previous year and from April-September of the current year (Fig. 7). Statistically significant (p<0.05) positive correlations between the annual height increment and the monthly mean minimum temperature in July of the current year were detected on most plots, except for N1-4200 (r = 0.18, p>0.05). On the whole, the annual height growth showed statistically significant positive correlations with the mean minimum summer temperature (from June to August) of the current year (Fig. 7). The corresponding correlation coefficients ranged from 0.4–0.6, except for the site N1-4200 (r = 0.33, p<0.001). Moreover, statistically significant positive correlations existed between the annual height increment at SE1 and the monthly mean maximum temperature of the previous December, ranging from −3.6 to 6.4°C based on a lapse rate of 0.75°C/100 m for the monthly maximum temperature [31]. With N1, such a significant correlation was confined to N1-3800. Negative effects of temperature on height increment were only observed at lower elevations N1-3800 and N1-4000, with significant negative correlations between the annual height increment and mean minimum temperatures in the previous or current August.

In all cases, precipitation showed only weak and rarely significant correlations with height increment in individual cases. Statistically significant correlations between height increment and precipitation were detected only in individual cases, for instance for the current January on SE1-4200 (r = −0.33, p<0.05), current February on SE1-3800 (r = −0.47, p<0.05), previous November on SE1-3800 (r = 0.46, p<0.05) and current April on SE1-4000 (r = 0.45, p<0.05).

## Discussion

A reduction in both maximum/mean tree height and mean annual height growth rate with increasing altitude is in agreement with reports from other areas (e.g. [32]–[34]). Maximum tree height is an important feature of forest vegetation because it is directly connected to local demographics, standing biomass and resource use [4], [17]. As suggested by population structure and maximum/mean tree growth, a decline of the above-ground forest biomass of Smith fir is evident with increasing elevation. However, further study is necessary to be able to use the maximum tree height as a quantitative tool for describing above-ground biomass in different altitudinal belts. The declining trend of maximum and mean height growth indicates how high-altitude environmental conditions (such as low temperature, low temperature-induced water stress, low nutrient availability and strong winds) affect important aspects of tree performance [18], [35]–[37]. In a modelling study, Kempes et al. (2011) were able to anticipate potential changes in maximum tree height as a result of changes in meteorology over a wide range of environments and tree species across the entire continental United States. The decline in the maximum and mean tree growth in our study may be driven by elevation-dependent temperature decline.

Maximum and mean tree height growth at different elevations (representing a temperature gradient) may provide a major insight into the biophysical determinants of maximum height and into the mechanism controlling tree-line formation, a topic for ongoing debate. Trees grow tall where resources are abundant, stresses are minor and competition for light places a premium on height growth [38]. Several studies have shown that environmental factors, especially low temperatures, at high altitudes restrict the physiological processes responsible for tissue formation, such as photosynthesis, respiration, allocation of food and shoot growth [18], [35], [36], [39]. As observed, plant height appears not to determine the freezing resistance of high-elevation plant species [40], [41]. From a physiological point of view, increasing leaf water stress due to gravity and path length resistance, as well as the conflicting requirements for water transport and water column safety, may ultimately limit leaf expansion and photosynthesis for further height growth, even with ample soil moisture [37]–[39], [42], [43]. In the Sygera Mountains, the wind is not strong enough to affect the height growth of Smith fir, as shown by a low average wind velocity (annual wind speed around 0.9 m/s) at the tree line [23] and the absence of wind-flagging trees [20]. The declining trend in height growth of Smith fir points to a stress gradient along the altitudinal transect, which is characterized by the dominant temperature gradient. Given the dominant role of water stress in limiting tree height growth [37]–[39], [42], [43], low temperature-induced water stress or water uptake [44]–[48], together with gravity, appears to play an important role in limiting tree height of Smith fir with increasing elevation. It was supported by an increasing trend of leaf δ^13^C values and a decreasing trend of the specific leaf area for Smith fir and *Rhododendron aganniphum* var. *schizopeplum* with increasing elevations the Sygera Mountains, indicative of increasing water stress [48], [49]. As reported, leave δ^13^C can even be used to predict maximum tree height [39]. The decreasing trend in the specific leaf area with increasing tree height or altitude is likely a mirror of a linear decrease in xylem water potential with gravity [50]–[52]. However, one or more ecological features (for example, minimum temperature, soil nutrients, periodic recruitment, allocation of food, disturbance and/or length of the growing season) probably further limit the ability of individuals at the highest altitudes to fully occupy the physical space [18], [51], [52].

The variation of maximum tree height at different elevations provides a clue to tree growth response to global warming in natural environments, via a space-for-time substitution. Height growth, in particular at elevations above 4200 m a.s.l., will benefit from warming as long as precipitation is not a growth-limiting factor. For example, the maximum/mean tree height at above 4200 m a.s.l. will increase by around 50% on a north-facing slope (Fig. 3), given an increase of mean summer temperature of 2.6°C, corresponding to a temperature difference between 3800 and 4200 m a.s.l. based on a lapse rate of −0.65°C/100 m [31].

Significant correlations among tree height, DBH and age suggest that both tree height and DBH can be excellent indicators of tree age, but with different regression slopes for different altitudes. On the other hand, it implies that the height increment at different elevations may be limited by climatic factors that also limit radial growth, such as the mean minimum July and summer mean temperatures [7]. Furthermore, pronounced correlations between tree height and age and the composition of height classes can reveal the age structure of each stand. However, the tree height-DBH-age relationship at low elevations cannot be used as an indicator for higher elevations. In comparison with radial growth [7], height increment series show more low-frequency variation, implying different performances between meristems in shoot buds and cambium and their interaction with the environment.

We recorded an overall increasing trend in height increment in past decades along the altitudinal gradient, which is in agreement with recent summer warming [19]. It has also been reported that the recent increase in height growth of tree line black spruce in northern forest-tundra sites in Canada is one of the registered responses to recent climate warming [8]. It is likely that future warming will enhance the height increment of Smith fir in different altitudinal belts. However, it must be cautioned that this may be partly related to tree age and size.

Our study shows that the mean minimum July and summer temperatures (from June to August) of the current growing season displayed a consistent and significant correlation with the annual height increment of Smith fir, regardless of topographical aspects or tree age along the altitudinal transects. As we expected, such climatic responses are in agreement with those of radial growth on the same sites [7] and with studies of other tree species and areas, which have reported a significantly positive effect of current summer temperature on shoot elongation (e.g. [8]). However, our findings do not agree with the findings of some other studies at high latitudes, in which the temperature of the previous summer was reported to be the dominant height-growth limiting factor of Scots pine (*Pinus sylvestris*) at the northern timberline in Fennoscandia (e.g. [12]–[14], [53], [54]) or of *Pinus pumila* in Central Japan [55]. Compared with high latitudes, stronger solar radiations on the Tibetan Plateau may shape a strategy that tree height growth is more sensitive to climate during shoot elongation. In addition, it has been reported that the annual height increment of Norway spruce (*Picea abies*) is fairly insensitive to climate in the southeastern European Alps [28].

As reported by James el al. (1994) [27], the shoot increment of Scots pine was primarily inhibited by mean temperatures below 6–8°C. Based on a lapse rate of 0.65°C [31], the mean and minimum temperatures in July and summer range from −0.1°C to +8.1°C at altitudes from 3,800 to 4,400 m a.s.l. in the Sygera Mountains. In such a case, we could expect height growth to be mainly limited by low temperature. Since the warmest month in the year, July, may be a critical time for the shoot growth of Smith fir, low temperatures in July are a pronounced limiting factor.

It is worth noting that the effects of the mean minimum summer temperature on shoot elongation of Smith fir did not show an increasing trend with ascending altitude, not supporting our hypothesis. The ratio of root biomass to aboveground biomass increased with increasing elevation [51], likely disrupting the relationship between climate and the height growth of Smith fir. Other environmental factors, such as low resource availability, and not only low temperatures, may partly overshadow the influence of the mean summer minimum temperature on shoot extension.

In addition to the importance of summer temperatures, at all SE1 stands and at N1-3800, the mean maximum temperature of the previous December showed a significant influence on the annual height growth. This indicates that higher temperatures in winter prior to the growth season positively affect the overwintering of the buds in Smith fir and thereby promote shoot extension during the next growth period.

Furthermore, our study shows that precipitation does not seem to be limiting for the height growth of Smith fir at different elevations, despite some exceptions with individual stands. This is in agreement with the results of tree-ring width analyses on the same sites [7]. Precipitation is perhaps not limiting because the annual sum of precipitation is relatively high, i.e., above 800 mm in the Sygera Mountains [23], [24].

### Conclusions

This study is the first survey of height increment and recent height growth trends with Smith fir as an example tree species, along different altitudinal belts on the southeastern Tibetan Plateau. The mean tree height, as well as the mean annual shoot elongation rate, declined with increasing altitude. Taking into account that two periods of development are involved in the shoot elongation of fir species, environmental conditions both in the previous and current year would affect the annual height increment of a tree. The mean minimum summer temperature (more precisely, the mean July minimum temperature) was the dominant limiting factor for shoot growth. Precipitation was apparently not growth-limiting in most study plots. The annual height increment, although showing the same response to climate as the radial growth at different elevations, could nevertheless be a supplementary proxy for dendroecological and dendroclimatic studies on the Tibetan Plateau. We observed an increasing trend in height growth on all sites, suggesting that a summer warming trend will enhance Smith fir's growth throughout its altitudinal distribution range. However, it should be noted that our data for shoot extension were confined to a short period of observation, precluding a robust understanding of height growth performance and its response to climate. In order to better understand the growth dynamics of a tree as a whole and its response to climate, future studies should include weekly monitoring of shoots and the radial increment of Smith fir trees within a growing season, as recommended by Rossi et al. (2009) and Seo et al. (2010) [56], [57]. For a better comparison with ring width, which is commonly used as an indicator of climate change at high-elevation sites, we are attempting to construct shoot-increment chronologies of Smith fir longer than 100 years at the upper timberline. It also raises the question for our future research of whether the maximum tree height is a useful ecological indicator to the above-ground biomass, perhaps together with the below-ground biomass of Smith fir at different elevations.
